# Studying Structural Details in Complex Samples. I. Combining two Chromatographic Separation Methods with Ultrahigh Resolution Mass Spectrometry

**DOI:** 10.1021/jasms.4c00226

**Published:** 2024-11-18

**Authors:** Jens Dreschmann, Lilla Molnarne Guricza, Wolfgang Schrader

**Affiliations:** Max-Planck-Institut für Kohlenforschung, Kaiser-Wilhelm-Platz 1, 45470 Mülheim a. d. Ruhr, Germany

**Keywords:** complex mixtures, deasphalted crude oil, 2D-chromatography, hyphenation, HRMS, structural elucidation

## Abstract

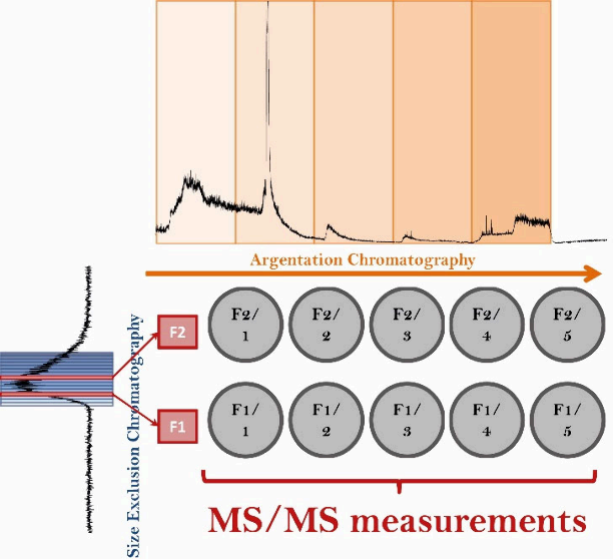

The analysis of complex mixtures poses a challenge due to the high number of compounds present in a mixture, which often exceed the capabilities of analytical methods and instruments. Even more challenging is understanding the structural details of compounds within a complex sample. Most analytical methods provide just bulk information on complex samples, and individual structural details cannot be observed. High-resolution mass spectrometry, the best method to analyze complex samples, suffers from inherent problems for structural studies in complex systems because collision-induced fragmentation (CID) measurements cannot provide data from individual compounds alone. The combination of different steps of chromatographic separation, here the combination of size exclusion chromatography with argentation chromatography, provides sufficient reduction in complexity to implement a method that allows gaining structural details of individual compounds within a complex mixture. The combination of offline size exclusion chromatography followed by online argentation chromatography effectively creates fractions based on the respective properties of the compounds in the mixture (size and number of π-bonds and heteroatoms) and reduces matrix effects to a great extent. Mass spectrometry with ultrahigh resolution provides basic chemical information for each detected compound and also provides the opportunity to gain structural information from MS/MS experiments. The results indicate effectively separated sample fractions yielded by the chromatographic steps with tremendously decreased total numbers of compounds. Especially, argentation chromatography proved to be a valuable separation tool when it comes to heteroatom-containing constituents. In the end, the fragmentation experiments indicated high-quality data due to the clean ion isolation enabled by prior separation. The structural elucidations provided deep insights into the carbon space of crude oil.

## Introduction

Many analytical fields, like proteomics, environmental sciences, or petroleomics, face the problem of highly complex samples. The quest for understanding single compounds amid tens of thousands of compounds is indeed comparable to looking for a needle in a haystack. Nevertheless, the objective of achieving structural elucidation in complex mixtures would sometimes be worthwhile. In case of an oil spill, for example, there are many crude oil constituents or their transformation products, which show the tendency of persistence.^1,2^ Little is known about these persistent organic pollutants (POPs) and their impact on ecosystems especially when it comes to high molecular weight polycyclic aromatic hydrocarbons (PAHs).^3^ The currently existing risk assessment of PAHs still refers to the 16 PAHs from the US EPA (United States Environmental Protection Agency) priority pollutant list.^4^ However, prior to the risk assessment toward toxicity and ecotoxicity and inclusion of single compounds within complex mixtures like crude oil, a characterization of these compounds is obligatory. Numerous approaches to deal with this complexity exist, and there is a full range of applications.^5−8^ However, very few methods deliver detailed information about the structural motifs of individual compounds. Conventional structure determination methods such as NMR or IR spectroscopy deliver detailed structural information about single compounds. Their limitations go along with increasing complexity of the sample since the provided information is bulk only. In contrast, mass spectrometry offers compound-resolved measurements and the opportunity of structural elucidation by performing fragmentation experiments (MS/MS or rather MS^n^).

The measurement of a complex sample via mass spectrometry can generate more than 100000 distinct signals which represent different chemical compositions.^9^ High resolving power, high mass accuracy, and high dynamic range are the requirements for the analysis of such complex mixtures.^10,11^ State-of-the-art instruments that operate on Fourier transform based techniques such as ion cyclotron resonance (ICR) and an Orbitrap mass spectrometer fulfill these premises.^12−18^ The data generated by this high-resolution mass spectrometer (HRMS) provide exact masses at a mass accuracy of far below 1 ppm and allow the precise assignment of the respective elemental compositions. Various ionization techniques (ESI, APCI, APPI, APLI)^19−23^ cover a broad range of component polarities and relativize ionization-induced ion suppression.^24,25^ Furthermore, different measurement modes lessen instrument related discrimination effects.^9^ However, the major drawback of mass spectrometry in this context is the limitation of exact masses. The bare elemental composition lacks structural information, since an exact mass always represents more than one possible structure. The ability to perform fragmentation experiments (product ion scan, etc.) on structures enables assumptions of the precursor ion’s structure. Nevertheless, it remains problematic to conduct these kinds of experiments on an untreated raw crude oil sample due to its complexity. The number of compounds per nominal mass makes it next to impossible to isolate single compounds with existing mass analyzers due to limitations in isolating individual signals within a small *m*/*z* range.

**Figure 1:**
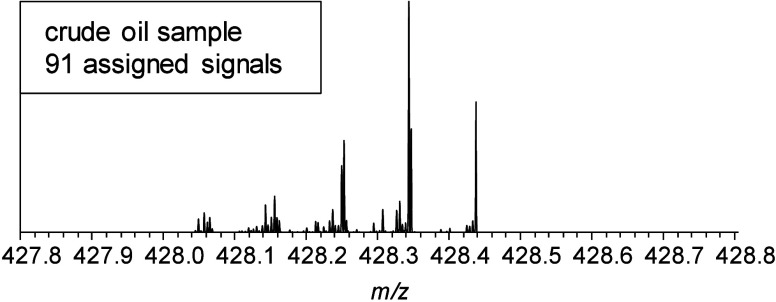
Scale-expanded mass view of a crude oil spectrum revealing 91 different signals in a mass range of less than 0.5 Da.

Crude oil, for example, can theoretically contain millions of compositional and configurational distinct chemical structures according to estimations.^9^ Up to date measurements can easily reveal sometimes more than 100 distinct signals per nominal mass (Figure 1).^26,27^ The reduction of ions per nominal mass or, in easier words, the simplification of the sample is the only logical and inevitable step to solve this issue.

Separation techniques like different types of chromatography enable fractionation of the sample, with fractions reduced in their total number of compounds present. The analysis of crude oil established a broad range of possible tools in the field of chromatography. Among many advantages of gas chromatography (GC), the low vapor pressure of higher mass compounds in crude oil impedes evaporation and, therefore, makes GC unsuitable for a universal simplification approach.^28^ In contrast, when using high performance liquid chromatography (HPLC) these problems of volatility are eliminated, but separation efficiency is reduced. HPLC suffers from lower peak capacities and solubility issues of the crude oil, especially when it comes to heavy crude oils, oil sands, and shales.^29^ Deasphalting of the sample separates the low soluble asphaltene fraction from the crude oil and provides a soluble fraction for HPLC.^30,31^ The lacking peak capacity can be compensated by a two-dimensional approach either as a comprehensive technique (LCxLC) or as an offline method. Offline approaches allow for operation with less equipment and offer the opportunity of sample enrichment. Moreover, comprehensive techniques are incapable of solvent changes between chromatographic dimensions, which restricts the first dimension run on nongradient conditions and increases the requirements of the mobile phase in two ways. First, a high solubility of the sample is categorical, but even the deasphalted crude oil makes this a challenge. The broad molecular weight distribution (ranging from 200 to ca. 1000 Da), wide range of boiling points, and differing polarities (from nonpolar to polar with mostly N, O, and/or S heteroatoms) and acidities (acidic, neutral or basic) explain these solubility issues.^32−34^ Second, the stationary phase requires the solvent to be inert toward the column material.

Different separation methods have been used to overcome these obstacles. For an example, ligand-exchange chromatography with a Pd(II)-ion-based stationary phase proved to be an effective technique for the separation of heteroatom-containing molecules such as polycyclic aromatic sulfur compounds.^35^ Other approaches utilize β-cyclodextrin,^36^ Ag(I)-mercaptopropyl bonded on silica gel,^37,38^ or even a dual approach with a 2,4-dinitroanilinopropyl silica (DNAP) column combined with a cation exchange column^39^ as the stationary phase which separate by the strength of the π-system. Another effective separation mechanism according to the molecular weight, size-exclusion chromatography (SEC), was successfully applied on crude oil fractions.^40^ Normal-phase liquid chromatography using a polar aminocyano stationary phase coupled to mass spectrometry effectively separated basic and nonbasic N heterocycles.^41^ Especially, the universal separation character of approaches such as SEC and argentation chromatography (ARG) emphasizes them as a formidable way to gain the most comprehensive view on crude oil and its fractions.

In any way, the HPLC separation of crude oil has to be understood as bulk separation rather than the separation of single compounds, even with the application of comprehensive techniques. However, the bare minimum objective for the investigation of single compound structures in a deasphalted crude oil is not a compound-by-compound separation but a reduction of signals per nominal mass to be as low as possible. This study focuses on the investigation of using different types of chromatographic separation—size exclusion chromatography and argentation chromatography—to reduce the complexity of a deasphalted crude oil as test sample. The target is to reduce the isobaric complexity until it is sufficient for subsequent fragmentation experiments. High resolving power is a key element for the analysis of bulk fractions and proper fragment identification; therefore, the application of HRMS is obligatory. The SEC is carried out in offline mode to allow the execution of several runs in order to preconcentrate the resulting fractions. The design for the separation by ARG and the analysis with HRMS is an online approach. This hyphenation enables the consecutive isolation and fragmentation of the compounds without losing the chromatographic resolution.

## Experimental Section

### Instruments and Methods.

Size exclusion chromatography was performed on an Agilent 1100 HPLC system (Agilent Technologies, Waldbronn, Germany) by injecting a 100 μL sample. Two subsequent-coupled styrene-divinylbenzene copolymer analytical columns (300 × 8.0 mm ID, 5 μm particle size) (PSS, Mainz, Germany) with pore sizes of 1000 and 100 Å were used for separation. THF was utilized as the mobile phase at a flow rate of 1 mL/min.

For argentation chromatography, a silver(I) mercaptopropyl silica based phase (5 μm particle size) was synthesized in our laboratory as described in previous studies.^37,38,42^ A stainless-steel column (250 × 2.0 mm ID) was wet-packed at 400 bar and conditioned with a mixture of toluene and chloroform (7:3, v/v). The measurement was performed using an UltiMate 3000 HPLC system (Thermo Fisher Scientific, Bremen, Germany) by injecting a 100 μL sample. A mixture of toluene and chloroform (7:3, v/v) was used as the mobile phase with stepwise increasing ratios of dimethyl sulfoxide (DMSO) according to the following gradient: 0% (0–10 min), 2% (10–20 min), 5% (20–30 min), 10% (30–40 min), 15% (40–50 min) and returned to 0% (50–70 min). The flow rate was set to 75 μL/min.

Mass spectra were recorded on a research-type Orbitrap Elite and a 7T LTQ FT Ultra FT-ICR mass spectrometer at a transient of 1.5 s (Thermo Fischer Scientific, Bremen, Germany). The mass range was recorded within 200–800 Da using full scan mode with a mass resolving power of 480000 or 200000 (fwhm at *m*/*z* 400) for the Orbitrap or ICR, respectively. Mass spectra were recorded over the time of the chromatographic run as follows. First, a full scan was recorded in the mass range of 200–800 Da. Then, preselected masses were isolated and subsequently fragmented at stepped collision energies. Parent ions were chosen previously based on their peak intensity to ensure a sufficient number of isolated ions for fragmentation. For each of these scan events, 7 micro scans were summed to provide adequate data depth.

Atmospheric pressure photoionization was performed in positive mode on a Krypton VUV lamp (Syagen Technologies, Tustin, CA, USA) with a photon emission at 10.0 and 10.6 eV. The heated sprayer was operated at 380 °C using nitrogen as sheath gas. Operating conditions for the instrument were as follows. APPI source conditions: vaporizer temp, 400 °C; sheath gas, 38; auxiliary gas, 15; sweep gas, 2; capillary temp, 275 °C; capillary voltage, 36 V; tube lens voltage, 125 V.

Collision-induced dissociation (CID) was used for fragmentation measurements, and the parent mass was selected with an isolation window of 0.8 Da. The isolated ions were recorded and then kinetically excited with medium (30%) and high (45–50%) energy (in the case of Thermo Scientific LTQ instruments, it is called normalized collision energy) using helium as collision gas.

### Sample Preparation

Deasphalted crude oil was prepared from a heavy crude oil from a North American source according to the first step of a modified IP 143 method.^43^ For direct infusion into the mass spectrometer, the deasphalted crude oil sample was diluted in a mixture of toluene and chloroform (7:3, v/v) to a concentration of 250 μg/mL, sonicated for 5 min, and subsequently analyzed without further treatment.

For SEC measurement, the deasphalted crude oil sample was diluted in tetrahydrofuran (THF) to a final concentration of 4 mg/mL and sonicated for 20 min. Fractions were collected in 30 s intervals from 14 to 34 min. Fifteen SEC runs were carried out, and the corresponding fractions were combined and evaporated to dryness.

Selected fractions were dissolved in a mixture of toluene and chloroform (7:3, v/v) resulting in a concentration of 2 mg/mL. Samples were separated online on an argentation chromatography phase coupled to a mass spectrometer and analyzed online. The postcolumn split flow (ratio of approximately 2.5:1) passed the UV detector with a flow rate of 55 μL/min and the mass spectrometer at a flow rate of around 20 μL/min.

### Data Analysis

The acquired data were analyzed by Xcalibur 2.2 software (Thermo Fisher Scientific, Bremen, Germany) and Composer V1.5.0 software (Sierra Analytics, Modesto, CA, USA). For peak assignments the following criteria regarding the number of possible elements and the number of double bond equivalent (DBE) were applied: 0 < H < 1000, 0 < C < 200, 0 < N < 3, 0 < O < 3, 0 < S < 3, and 0 < DBE < 40 with a maximum mass tolerance of 1 ppm.

During the evaluation, DBE is used as an important parameter for comparison of the unsaturation degree of the components. DBE refers to the sum of the rings and the number of double bonds within a molecule and is calculated from the molecular formula (C_*c*_H_*h*_N_*n*_O_*o*_S_*s*_) of the assigned individual peaks by the equation
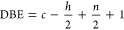


## Results and Discussion

**Figure 2:**
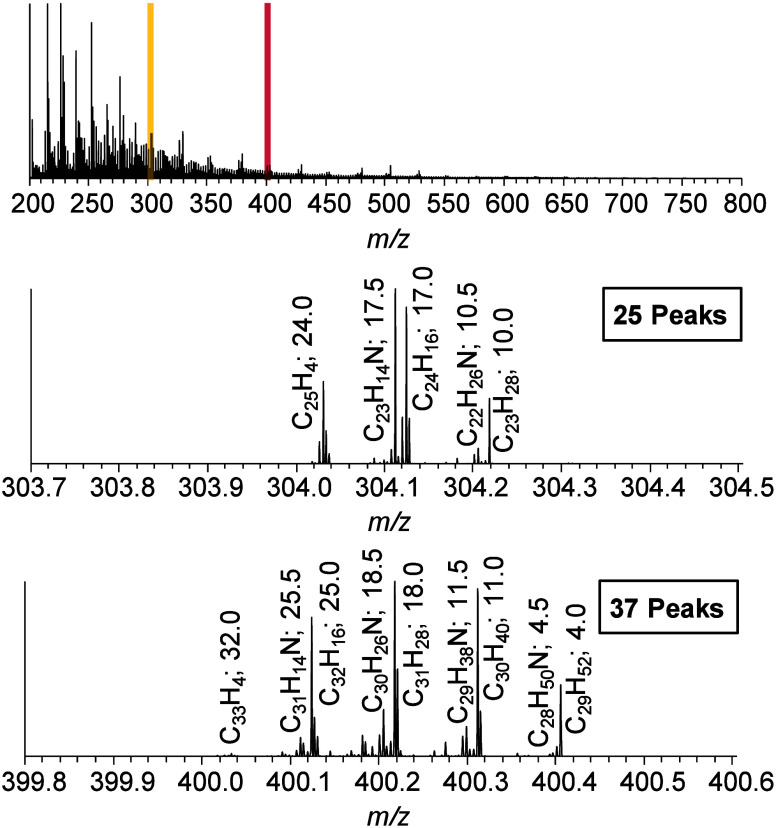
Full scan mass spectrum of a deasphalted crude oil sample in the range of 200–800 Da (top). Scale-expanded mass views of 0.8 Da around 304 and 400 Da with the most abundant peaks named by their assignments and their DBE values (middle and bottom). The integer shows the radical form of the ion while the noninteger gives its protonated species.

The enormous complexity of deasphalted crude oil is illustrated by the mass spectrum in Figure 2 (top). Each signal covers one chemical composition with at least one structural configuration. Mass spectra in the middle and at the bottom of Figure 2 show isolation windows for deasphalted crude oil, which are typically applied at a 0.8 Da width.

This example shows two different small mass windows at *m*/*z* 304 and 400 that contain 25 and 37 assigned signals, resepectively. Depending on the oil, there are different fractions of heteroatoms (N, O, and S), further increasing the number of possible elemental compositions and the complexity. Looking at the assignments in Figure 2 indicates mostly hydrocarbons (C_25_H_4_, C_24_H_16_, C_23_H_18_, etc.) and nitrogen-containing protonated ions (C_23_H_14_N, C_22_H_26_N, C_31_H_14_N, etc.) in a DBE range of 4–32. This matches the expectations since the investigated deasphalted crude oil originates from a nitrogen-rich oil and the ionization method used (APPI) predominantly creates radical ions but also produces protonated molecules. Dopant-assisted ionization by toluene might also take place and may lead to either radical or protonated species.^22^ The isolation and subsequent fragmentation of that amount of compounds with a high level of diversity would lead to an unanswerable fragmentation pattern.

Another problem of such a mass spectrum or mass spectrometry, in general, is the lack of resolving isomeric structures. Each signal contains an unknown number of isomeric variants, which makes the resulting fragmentation pattern even harder to decipher. Altogether, this indicates how easily complex mixtures exceed the capabilities of fragmentation experiments for structural investigations of single compounds. Thus, the first step prior to structural investigations is the reduction of complexity, or rather the use of separation techniques.

**Figure 3:**
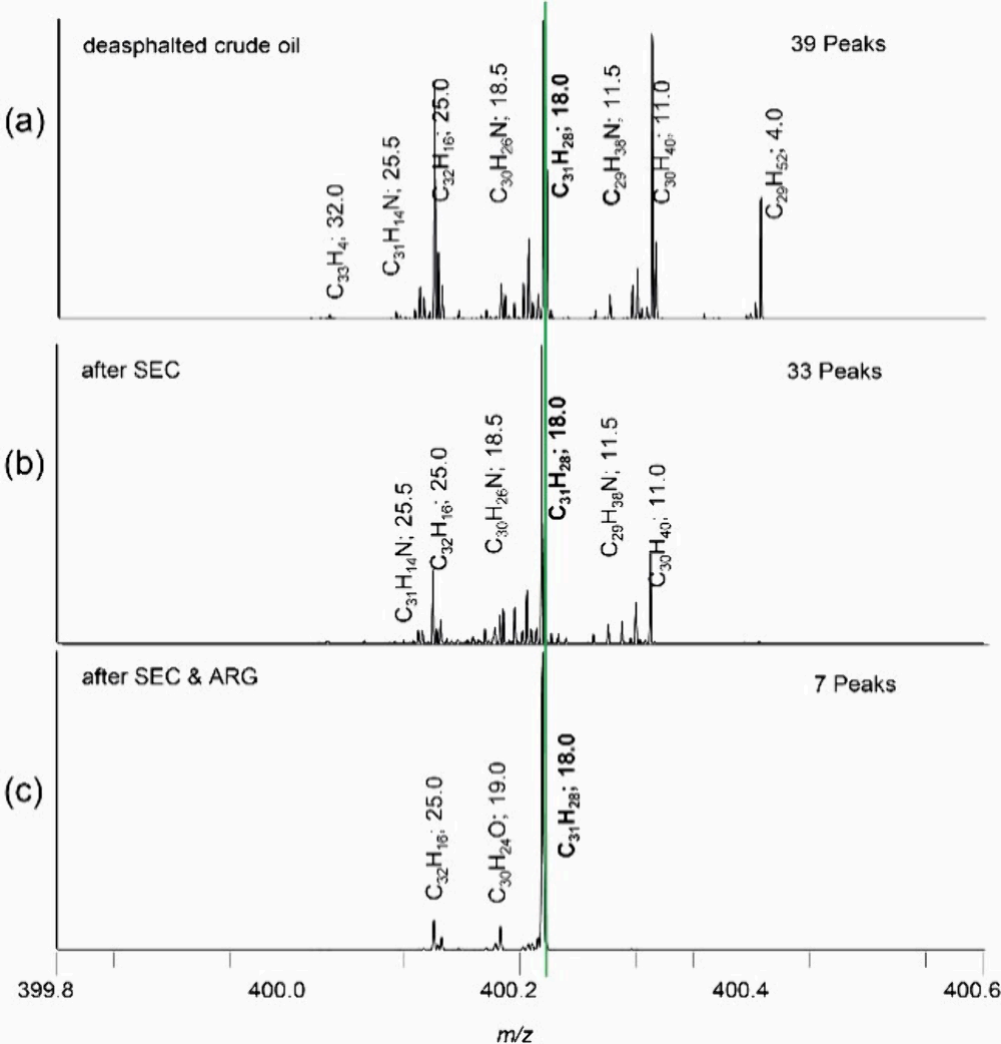
Isolation windows of (a) a maltene sample, (b) a SEC fraction (center), and (c) the SEC fraction during argentation chromatography at 0% DMSO (bottom). The mass range of 399.8–400.6 Da is shown.

The broad range of different properties in deasphalted crude oil makes it impossible to avoid a secondary separation dimension. Previous investigated chromatographic techniques in single dimension separation still indicated high numbers of ions per nominal mass.^42,44^ However, the combination of size and structure related first dimension and a π-system-based separation in the second dimension achieves a significant drop of compounds within an isolation window of 0.8 Da (Figure 3).

The spectrum of the deasphalted crude oil shows 39 assigned compounds in a mass window of about 0.5 Da. After the SEC this number decreases to 33 assigned signals and ultimately ends at 7 assigned compounds after SEC and ARG.

The compounds shown here in the isolation windows of the deasphalted crude oil, SEC fraction 15, and 0% DMSO ARG fraction of this SEC fraction correspond to hydrocarbons (C_32_H_16_, C_31_H_28_, C_30_H_40_, C_29_H_52_) and mostly nitrogen-containing compounds as expected (C_31_H_14_N, C_30_H_26_N, C_29_H_38_N, C_28_H_50_N). The size-exclusion step led to the complete depletion of low DBE compound signals (C_29_H_52_, C_28_H_50_N). Furthermore, the signals next to C_31_H_28_ indicate reduced signal intensity to about a half compared to the deasphalted crude oil (C_32_H_16_, C_31_H_14_N, C_30_H_40_, and C_29_H_38_N). The explanation hides in the structures of these ions, since they show similar masses but do not share the same structures. Compounds with lower DBE values point to smaller cyclic paraffinic or aromatic cores with longer alkyl chains attached. Examples are the elemental compositions of C_30_H_40_ and C_29_H_38_N. The second type of compounds appearing at similar retention times are showing a different type of structure, as here the carbon atoms are not present in longer aliphatic side chain but are used to form larger aromatic core system with higher DBE values such as C_32_H_16_ and C_31_H_14_N. Compounds with really small aromaticity and DBE values below DBE 10 are eluting later as they are filtered out in this example. Larger aromatic types are interacting less and appear to elute quickly, as they are usually of larger size. The difficulty lies in the intermediate types of compounds that have a smaller aromatic core but longer side chains. Here, these carbon atoms can be arranged in more than one side chain, leading to different types of structures that can be separated on a SEC phase. We can observe the results of this effect as only some of these intermediate sized compounds appear in the isolation window, leading to a reduced complexity, as was the intention for these experiments.

**Figure 4:**
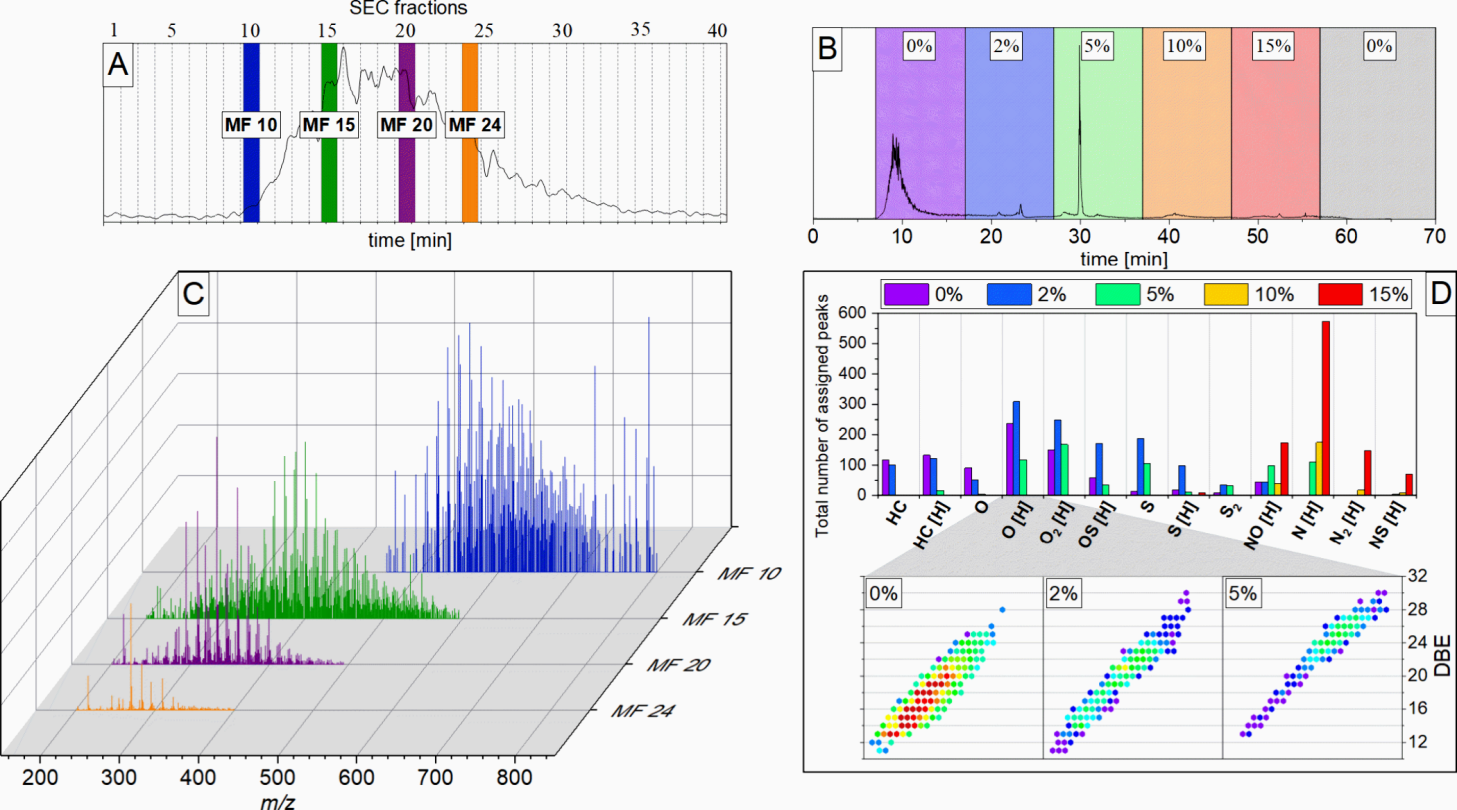
(A) Total ion chromatogram (TIC) of size-exclusion chromatography applied to deasphalted crude oil discriminated into 30 s segments. Blue, green, purple, and orange bars show the selected SEC fractions. (B) TIC of argentation chromatography applied to a SEC fraction divided into different fractions according to their DMSO ratio. (C) Averaged full mass spectra of SEC fractions 10, 15, 20, and 24 recorded during argentation chromatography (fraction 0% DMSO). (D) (top) Bar chart distribution appointing different compound classes of MF 20 (HC, hydrocarbon; O, S, and N, heteroatom) against their total number of assigned peaks. The color indicates the different DMSO ratios. (bottom) Kendrick plots of the O[H] class arranged by its DBE value (*y*-axis) and its mass-to-charge ratio (*x*-axis). The color encodes the monoisotopic signal (from red (=high) to blue (=low)).

For this study, the mass spectra of a few selected fractions (MF 10, MF 15, MF 20, and MF 24 (Figure 4A)) of the size exclusion chromatography (SEC fraction) were chosen to demonstrate this overlap (Figure 4C). Nevertheless, the mass distributions of these spectra indicate a mass shift from higher to smaller masses from MF 10 to 24. The ARG fraction exhibits a clear drop in the number of ions present (Figure 3 (bottom)). The most intense signals correspond mainly to two hydrocarbon compounds (C_31_H_28_, C_32_H_16_) and an oxygenic constituent (C_30_H_24_O). The absence of nitrogen-containing compositions is most striking at this point, especially when it comes to C_30_H_26_N with a DBE of 18.5. The most intense signal of hydrocarbon C_31_H_28_ shows a similar DBE value of 18.0, which raises the question of why these two molecules undergo a separation.

Argentation chromatography enables separation by π-systems, and the strength of the π-system can be measured by the amount of double bonds and ring structures. The DBE value correlates the number of double bonds and ring structures with the number of H atoms. Plotting the DBE against the molecular mass indicates the separation by π-systems (Figure 4D (bottom)). The higher the DBE value, the larger the π-system. The signal intensity shifts toward higher DBE values and molecular masses from a 0% to 5% DMSO ratio. This indicates a stronger retention for the stronger π-systems. However, the content of heteroatoms eventually has a stronger influence on the separation (Figure 4D (top)). The elution follows the order of pure hydrocarbons and oxygen-, sulfur-, and nitrogen-containing molecules (HC < O < S < N).

Two options are valid to explain this behavior. The first option is the difference in which heteroatoms affect π-systems by inductive and mesomeric effects. The second and more likely option is heteroatom complexation of the Ag(I)-ions. This explanation corresponds to the elution order since nitrogen-based ligands show stronger interactions with Ag(I)-ions than sulfur- and oxygen-containing compounds. This also explains the absence of nitrogenic compounds after ARG in Figure 3 (bottom).

In conclusion, the combination of both chromatographic approaches significantly decreased the complexity of the sample. Furthermore, the consecutive setup of two chromatographic dimensions provides the possibility of performing fragmentation experiments.

**Figure 5:**
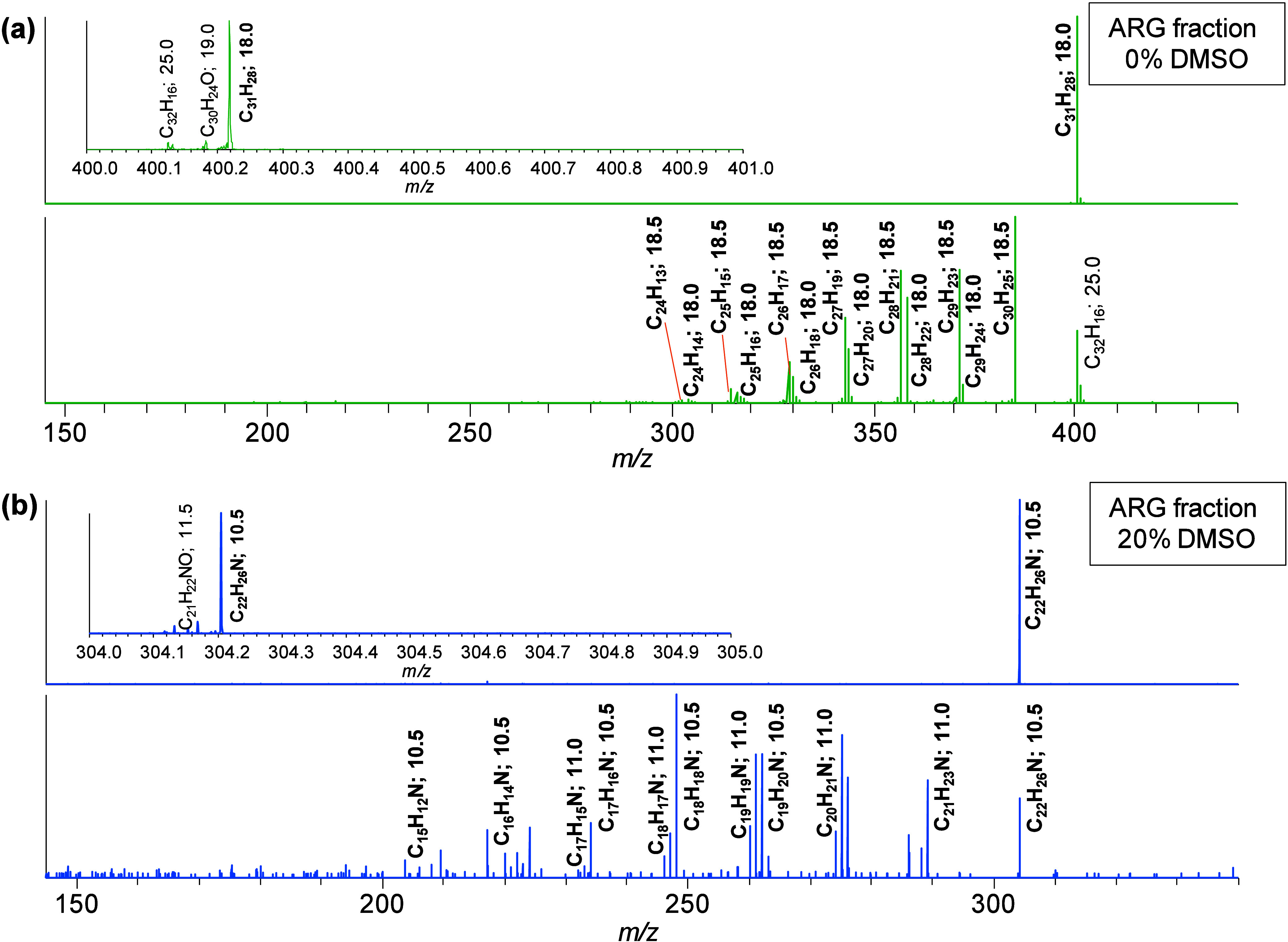
(a) CID isolation (top) and fragmentation (bottom) mass spectra of molecular ions of *m*/*z* 400 ± 1 with collision energies of 0% and 35%, respectively. The spectra shown originate from ARG fraction 0% DMSO of SEC fraction 15. The small window on the top left position indicates a 1 Da narrow-band scan of the mass range *m*/*z* 400–401. (b) CID isolation (top) and fragmentation (bottom) mass spectra of molecular ions of *m*/*z* 304 ± 1 with collision energies of 0% and 45%, respectively. The spectra shown originate from ARG fraction 15% DMSO of SEC fraction 15. The small window on the top left position indicates a 1 Da narrow-band scan of the mass range *m*/*z* 304–305.

Arbitrarily chosen nitrogen-containing (*m*/*z* 304.2060) and hydrocarbon (*m*/*z* 400.2186) compounds are shown to demonstrate the fragmentation behavior (Figure 5). The nitrogenic compound elutes in the 15% DMSO fraction, while the hydrocarbon elutes in the 0% DMSO fraction of the same run. The spectra display the related isolation and MS^2^ scans (Figure 5a,b) for both compounds. The two spectra on top of each example show the isolation windows of the isolated ions prior to fragmentation. There are two assigned peaks in the mass range of *m*/*z* 304–305 corresponding to formulas with elemental compositions of C_22_H_26_N and C_21_H_22_NO, though the latter is only present in small intensity. The influence on the fragmentation is negligible due to the small intensities of the accompanying ions. The salient fragment peaks indicate losses of methyl (CH_3_, 15 Da), ethyl (C_2_H_5_, 29 Da), propyl (C_3_H_7_, 43 Da), butyl (C_4_H_9_, 57 Da), pentyl (C_5_H_11_, 71 Da), hexyl (C_6_H_13_, 85 Da), and heptyl (C_7_H_15_, 99 Da) groups for the hydrocarbon compound and up to pentyl group losses for the nitrogen-containing structure. The constituted loss of an alkyl chain, for example, a methyl or ethyl group by homolytic cleavage, leads to an increase of DBE by half a unit (18.0 → 18.5 or 10.5 → 11.0). These methyl or ethyl fragments indicate the presence of an ethyl or rather a propyl side chain on an aromatic core. It has been shown in previous studies that benzyl cleavages are the dominant fragmentation mechanisms of alkyl chains attached to aromatic cores.^45^ This fragmentation is favored due to the formation of a stabilized tropylium ion. Therefore, such small fragments are not the result of a larger alkyl chain fragmenting into multiple aliphatic moieties but rather indicate isomers of different side chains, where each side chain produces only one distinct fragment. Alternative fragmentations by a McLafferty rearrangement lead to fragment losses of ethene, propene, or other alkenes. This heterolytic cleavage leads to no changes in the DBE value (18.0 → 18.0 or 10.5 → 10.5). This type of fragmentation can explain fragment losses of ethane (28 Da, C_2_H_4_), propene (42 Da, C_3_H_6_), butane (56 Da, C_4_H_8_), pentene (70 Da, C_5_H_10_), hexene (84 Da, C_6_H_12_), and heptene (98 Da, C_7_H_14_) for the hydrocarbon structure as well as for the nitrogenic composition.

**Figure 6:**
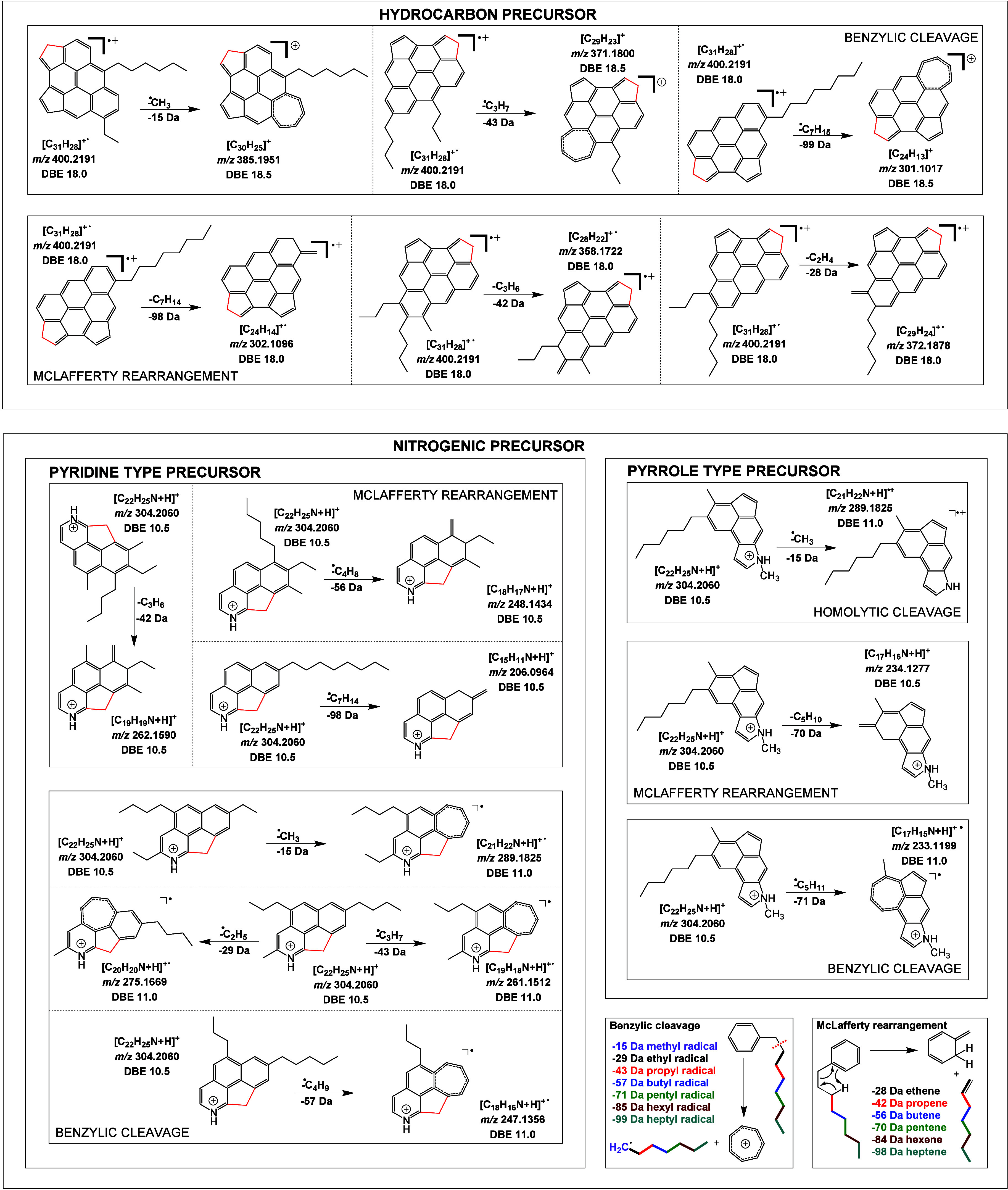
Possible proposed structures for a hydrocarbon compound of the 0% DMSO ARG fraction (SEC F15) (top) and for a nitrogen containing composition of the 15% DMSO ARG fraction (SEC F15) under different fragmentation mechanisms (bottom).

The fragment losses indicate an aromatic core structure of DBE 18 or rather 18.5 with, in total, eight carbon atoms in aliphatic side chains for the hydrocarbon structure (Figure 5 (top)). Possible proposed structures are suggested in Figure 6 (top).

The construction of these structures follows the intention to represent the most stable molecule configurations since crude oil underwent harsh conditions during its million years of genesis. These conditions merely allow nonreactive and stable compounds to exist. Different arrangements of aromatic rings might be possible as well as different positions and numbers of aliphatic chains. As seen in Figure 6 (top), in these examples the aromatic core contains at least one sp^3^-carbon (bond shown in red) since the elemental composition disallows a completely conjugated structure. Nevertheless, the assembly of two five-rings instead of one six-ring is more likely since this isomer offers an additional sp^2^-carbon and therefore provides a higher stability for the molecule. Recently, theoretical calculations have shown that conjugated sp^2^ systems with 5-membered rings are more stable than deficient systems that include either sp- or sp^3^-carbon.^46^ The positioning shown here was chosen arbitrarily and cannot be concluded from the present data. Nonetheless, there are some tendencies for the positioning of the alkyl chains. The intensities imply the benzylic cleavage compliant with the literature as the dominant reaction mechanism. However, the ratio of the benzylic cleavage and the McLafferty rearrangement fragments approaches an increasing alkyl chain length. This change suggests that the McLafferty rearrangement is taking place more frequently. The explanation may be that the branching and the length of the alkyl chains, as well as the number of alkyl chains and their positioning, play an important role in the fragmentation. On one hand the geometry of branched alkyl chains sterically impedes the proton transfer during the McLafferty rearrangement. On the other hand, it can be assumed that the more branched the alkyl chain is, the more it tends to form stable radicals due to inductive effects. Thus, the McLafferty rearrangement is still preferred over the benzylic cleavage since the radical molecular ion is present at a stabilized state, and this prolongs the available time span for the reaction. Vice versa, this means that the less branched an alkyl chain is, the less it tends to form stable parental radical ions and, therefore, favors the homolytic benzylic cleavage.

Two or more neighboring alkyl chains would probably show a similar behavior; the proton transfer is on a temporal perspective delayed due to steric reasons, but the inductive effect stabilizes the parental radical ion and, thus, the McLafferty rearrangement is favored. In conclusion, the intensities of the hydrocarbon fragments imply shorter, less branched alkyl chains, which share no close proximity. Especially, the attachment of ethyl, propyl, and butyl chains seems dominant.

The aromatic core of the nitrogenic composition counts a DBE of 10.5 and in total 6 carbon atoms in saturated side chains (Figure 5, bottom). The interpretation of the nitrogen-containing structure is a lot more sophisticated since the assembly of heteroatoms introduces additional variants (Figure 6 (bottom)). The configuration of the nitrogen holds either a pyrrole or a pyridine type compound. The pyrrole option enables the possibility of attaching alkyl chains to the nitrogen atom and allows fully conjugated core structures, while the pyridine option comes with at least one sp^3^-carbon. The example in Figure 6 (bottom) has the bonds to the sp^3^-carbon shown in red for a better understanding of the discussion.

**Figure 7:**
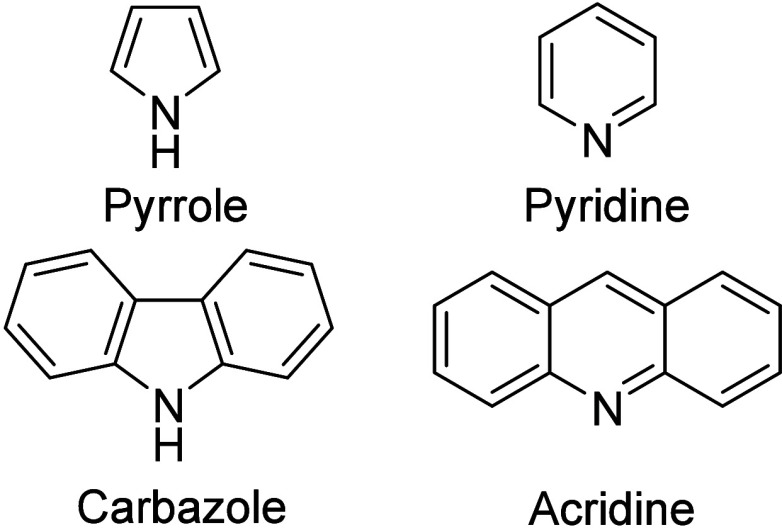
Structures of different nitrogen species.

Therefore, the pyrrole-type structure likely indicates better long-term stability in this case. Analytical standards of acridine (pyridine-type) and carbazole (pyrrole-type) showed coelution during the argentation chromatography. The structures are shown in Figure 7. Therefore, from a chromatography point of view, both options are possible, but since sp^2^-carbon builds up the lowest-energy structures, it is more likely to have pyrrole type compounds at hand instead of pyridine types. In any event, the possible proposed molecules contain either one or no sp^3^-carbon depending on the nitrogen configuration. Obviously, the intensity distribution features differences in contrast to the hydrocarbon compound. First, fragments generated by McLafferty rearrangement dominate in terms of intensity, the same as they did for the hydrocarbon precursor. Thus, the core structure probably exhibits neighboring and branched alkyl chains attached to the aromatic part of the structure. Second, the distinctive fragment peaks indicate pentyl, butyl, and propyl chains. The benzylic cleavage produces mainly methyl, ethyl, and propyl radicals, indicating ethyl, propyl, and butyl side chains for those structures. However, another homolytic cleavage reaction competes with the benzylic cleavage. In the case where pyrrole is alkylated, the homolytic cleavage can take place directly at the nitrogen atom forming a nitrogen radical. There is no information whether either benzylic or homolytic cleavage at the nitrogen atom is dominant. In summary, the cleaved alkyl chains share mostly a branched character, neighboring other alkyl chains. Furthermore, these alkyl chains contain more carbon atoms, in relation to the hydrocarbon structure discussed before.

## Conclusion

This study successfully demonstrated the utilization of combined separation techniques hyphenated to HRMS in order to perform MS/MS measurements on a complex sample such as crude oil. Size and weight related separation yield significantly less complex samples and set the basis for further separation. The π-system-based separation of deasphalted crude oil indicated an additional effective heteroatom-based separation. This 2D chromatographic setup remarkably reduced the number of ions per nominal mass. In consequence, the fragmentation experiments enabled the elucidation of basic structural motifs. The benyzlic cleavage and McLafferty arrangement appeared to be the main fragmentation mechanisms. Inferences from these mechanisms led to the assumption that the fragmented structures rest upon an aromatic core structure with alkyl chains attached to it. The precise positioning of these alkyl chains at the core fails elucidation without consecutive fragmentation (MS^n^). Nevertheless, the fragmentation already revealed coexisting isomers of these island-type compounds. Altogether, this approach provided detailed structural information on unique compounds within an extremely complex sample. Future exploitation of other crude oil fractions or even different complex mixtures seems possible.
